# Artefact and ablation performance of an MR-conditional high-power microwave system in bovine livers: an *exvivo* study

**DOI:** 10.1186/s41747-019-0115-4

**Published:** 2019-09-23

**Authors:** Antonia Grimm, Moritz Winkelmann, Jakob Weiß, Georg Gohla, Gunnar Blumenstock, Konstantin Nikolaou, Stephan Clasen, Rüdiger Hoffmann

**Affiliations:** 1 Department of Diagnostic and Interventional Radiology, University Hospital of Tübingen, Hoppe-Seyler-Strasse 3, 72076 Tübingen, Germany; 2 Institute for Clinical Epidemiology and Applied Biometry, University Hospital of Tübingen, Silcherstraße 5, 72076 Tübingen, Germany

**Keywords:** Ablation techniques, Cattle, Liver, Magnetic resonance imaging, Microwaves

## Abstract

**Background:**

We evaluated a magnetic resonance (MR)-conditional high-power microwave ablation system.

**Methods:**

An *ex* *vivo* 1.5-T evaluation was conducted by varying the sequence (T1-weighted volume interpolated breath-hold examination, T1w-VIBE; T1-weighted fast low-angle shot, T1w-FLASH; T2-weighted turbo spin-echo, T2w-TSE), applicator angulation to *B*_0_ (*A*-to-*B*_0_), slice orientation, and encoding direction. Tip location error (TLE) and artefact diameters were measured, and influence of imaging parameters was assessed with analysis of variance and post hoc testing. Twenty-four *ex* *vivo* ablations were conducted in three bovine livers at 80 W and 120 W. Ablation durations were 5, 10, and 15 min. Ablation zones were compared for short-axis diameter (SAD), volume, and sphericity index (SI) with unpaired *t* test.

**Results:**

The artefact pattern was similar for all sequences. The shaft artefact (4.4 ± 2.9 mm, mean ± standard deviation) was dependent on the sequence (*p* = 0.012) and the *A*-to-*B*_0_ (*p* < 0.001); the largest shaft diameter was measured with T1w-FLASH (6.3 ± 3.4 mm) and with perpendicular *A*-to-*B*_0_ (6.7 ± 2.4 mm). The tip artefact (1.6 ± 0.7 mm) was dependent on *A*-to-*B*_0_ (*p* = 0.001); TLE was -2.6 ± 1.0 mm. Ablation results at the maximum setting (15 min, 120 W) were SAD = 42.0 ± 1.41 mm; volume = 56.78 ± 3.08 cm^3^, SI = 0.68 ± 0.05. In all ablations, SI ranged 0.68–0.75 with the smallest SI at 15 min and 120 W (*p* = 0.048).

**Conclusion:**

The system produced sufficiently large ablation zones and the artefact was appropriate for MR-guided interventions.

## Key points

- An MR-conditional high-power microwave system was assessed for artefact and ablation performance in bovine *ex* *vivo* livers at 1.5 T.
- The applicator artefact is precise concerning tip depiction.
- Largest artefact diameters were measured with a T1-weighted gradient-echo fast low-angle shot sequence.
- Applicator angulation perpendicular to *B*_0_ causes largest artefact diameters.
- The MR-conditional microwave system provided appropriate dimensioned ablation zones.

## Background

Percutaneous thermoablation is an established minimally invasive treatment option for patients with hepatic malignancies who are not suitable for surgical resection due to impaired hepatic function, comorbidities, or unfavourable anatomic conditions [1, 2]. With radiofrequency (RF) ablation being the most common representative in the “toolbox” of local therapies, studies have shown that tumour location close to large vessels and size greater than 3 cm are critical factors regarding local recurrence-free survival [3–5].

Over the last decade, microwaves (MW) were introduced as an energy source and MW ablation (MWA) has developed into an alternative to RF ablation, with theoretical physical advantages and an increasing variety of commercially available MWA systems [6, 7]. Indeed, in comparison to RF ablation, MWA is not limited by desiccation around the applicator, allowing for higher ablation temperatures in larger ablation zones in a shorter time with a single applicator [8, 9]. Furthermore, studies have shown that MWA is less susceptible to the heat-sink effect of larger hepatic vessels [10]. A recent meta-analysis has shown lower local recurrence rates after MWA of larger tumours in comparison to RF ablation [11].

Besides a satisfactory ablation technique, precise applicator placement and therapy monitoring are relevant for successful thermoablation. In this respect, magnetic resonance (MR) as a guidance modality offers several advantages including an assessment of the ablation zone without application of contrast agent, free selection of imaging planes, temperature measurement with MR thermometry and radiation-free near real-time fluoroscopic sequences [12–14]. The high sensitivity of MR imaging (MRI) for small liver lesions is advantageous during tumour targeting and visibility of the smallest target tumours can be increased for hours by administering gadoxetic acid [15–18].

Despite these technical advantages, restricted availability of suitable scanners and higher costs are critical points in view of the relatively long durations of MR-guided procedures, so that MRI as guidance modality for thermal ablation is currently limited to specialised centres [19]. In this context, the introduction of an MR-conditional high-power MWA system might combine the advantages of both techniques and increase the significance of MR-guided thermoablation in the future by decreasing the procedure duration. A prerequisite for successful MR-guided MWA is a reliable and adequate configuration of the applicator artefact with an accurate depiction of the tip. Large antenna artefacts cause a good visualisation of the applicator, however, may impair the assessment of the ablation zone, so that a balanced artefact configuration is mandatory.

Thus, the purpose of this study was the preclinical evaluation of a new MR-conditional high-power MWA system regarding ablation performance and applicator artefact appearance.

## Methods

### Microwave tissue ablation technique and equipment

The experiments were conducted with a high-power MWA system with a maximum generator power of 150 W. The system was equipped with a pump for perfusion-cooling of the applicator shaft. The MW generator (ECO-100E2, Nanjing ECO Medical Instrument Co., China) worked at a frequency of 2.45 GHz. All experiments were conducted with a 14-G MW applicator (ECO-100AI13C, Nanjing ECO Medical Instrument Co., China) with a shaft length of 15 cm. The applicator is composed of a shaft consisting of titanium alloy and a ceramic tip with a length of 18 mm. A 4-m long coaxial cable connects the antenna with the generator enabling the generator to be positioned safely outside the MR scanner room during ablation.

### MRI protocol and artefact evaluation

Artefact evaluation was conducted in a 1.5-T short bore scanner (Magnetom ESPREE, Siemens Healthineers, Erlangen, Germany) with a horizontal main magnetic field (*B*_0_) and a four-channel body-array surface coil. The MW applicator was placed in an MRI phantom consisting of a Plexiglas box filled with a 0.2% gadolinium solution (Gadovist, Bayer Healthcare, Berlin, Germany). The phantom was positioned at the magnet isocentre and enabled a deflection of the applicator relative to *B*_0_ between 0° and 90°. The measurements were performed with three different sequences:

1. A three-dimensional T1-weighted volume interpolated breath-hold examination (T1W-VIBE) with chemically selective fat-saturation pulse, performed with flip angle of 10°, repetition time (TR) of 6.2 ms, echo time (TE) of 1.61 ms, bandwidth of 457 Hz/pixel, slice thickness of 1 mm, field of view (FOV) 192 × 192 mm, acquisition matrix 192 × 192, and reconstruction matrix 192 × 192;
2. A two-dimensional T1-weighted, fast low-angle shot (T1W-FLASH) gradient-echo sequence with periodic chemically selective fat-saturation pulses and flip angle of 70°, TR of 122 ms, TE of 4.36 ms, bandwidth of 139 Hz/pixel, slice thickness 4 mm, FOV 192 × 192 mm, acquisition matrix 192 × 192, and reconstruction matrix 192 × 192;
3. A two-dimensional T2-weighted turbo spin-echo (T2W-TSE) sequence with TR of 3750 ms, TE of 129 ms, flip angle 145°, echo train length 29, bandwidth 260 Hz/pixel, slice thickness 4 mm, FOV 192 × 192 mm, acquisition matrix 192 × 192, reconstruction matrix 384 × 384.

The following factors were systematically varied: sequence type (T1W-VIBE, T1W-FLASH, T2W-TSE), applicator orientation to *B*_0_ (0°, 45°, 90°), slice orientation with respect to the applicator (axial, coronal, sagittal), and encoding direction (phase encoding direction or frequency encoding direction being perpendicular to the long axis of the applicator) resulting in a total of 36 artefact measurements. Artefact analysis of the acquired images was performed with the open-source software ImageJ (http://rsb.info.nih.gov/ij). Artefacts were defined according to the American Society for Testing Materials as deviation of ± 30% from the median signal intensity around the applicator [20]. Imaging analyses were conducted in consent of two readers (AG and RH).

The artefact diameters were measured at the applicator tip and the applicator shaft. The tip location error (TLE) was assessed on images with coronal or sagittal orientation in relation to the applicator. The TLE describes the deviation from the measured distance between the distal end of the tip and the Plexiglas model and the actual set 10 mm distance. A positive TLE correlates with an overestimation of the applicator position in the long axis direction [21].

### Ablation protocols and ablation zone evaluation

All ablations were performed *ex* *vivo* at room temperature using three fresh bovine livers (Bos Taurus) obtained from a local abattoir. Before positioning of the MW applicator, large hepatic veins were explored with a metal probe to avoid close positioning. The ablation durations were 5, 10, and 15 min (maximum recommended duration). Ablations were conducted with a power of 80 W and 120 W (maximum recommended power for liver ablation with a 14-G antenna). Each combination was repeated four times resulting in a total of 24 ablations. Results were only made available to the manufacturer after completion of the experiments.

**Fig. 1:**
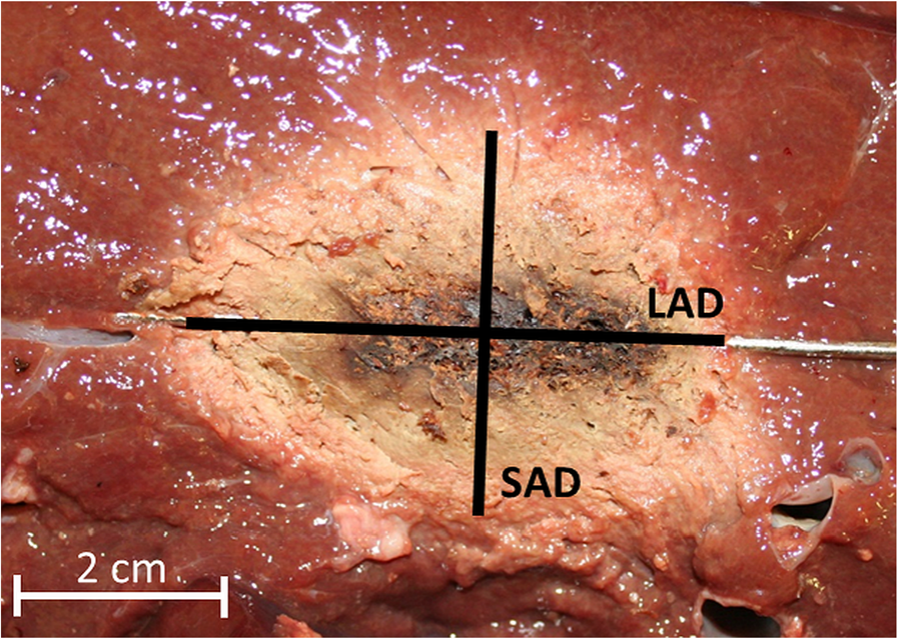
Cross-section of an ablation zone after 15 min ablation at 120 W. The long axis diameter (LAD) and short-axis diameter (SAD) are shown

After ablation, the antenna was replaced by a metal bar serving as guidance to dissect the liver along the ablation zone. For further measurements, the ablation zone was photographed (Canon, EOS 350D, Tokyo, Japan). The ablation zone diameter along the antenna insertion axis was defined as long-axis diameter (LAD). The largest diameter of the ablation zone perpendicular to the LA was defined as short-axis diameter (SAD) (Fig. 1). Dimensions were determined using callipers by measuring the perimeters of the white coagulation zone. The volume of the ablation zone was calculated using the ellipsoid formula for diameters (Volume = π/6*LA*(SAD)^2^). The shape of the ablation zone was determined by calculating the sphericity index (SI) = SAD/LAD.

### Statistical analysis

Acquired data were analysed with the statistical software JMP 13 (SAS Institute, Cary, NC, USA). To compare the TLE, shaft artefact diameter and tip artefact diameter in terms of sequence type, angulation to *B*_0_, slice orientation and encoding direction analysis of variance (ANOVA) was performed. The assumptions of variance homogeneity and normal distribution were checked. In the case of heterogeneity of variances, the Welch ANOVA test was used. If a significant overall effect was found, post hoc between-group comparisons were performed following the closed testing procedure and by using the Student *t* test [22, 23]. In case of comparing only two parameters, the Student *t* test was used instead of ANOVA.

To compare the effect of a different ablation power on the SAD, volume, and SI with respect to the ablation duration (5, 10, and 15 min) unpaired *t* tests was used. Results were displayed as mean ± standard deviation (SD). A *p* value < 0.05 was considered statistically significant for all tests.

## Results

### Applicator artefact

At all acquired sequences, the applicator presented a similar artefact pattern with a prominent shaft and a smaller tip artefact, distal 18 mm of the applicator. The appearance of the applicator was homogenous along the shaft and the tip without the appearance of blooming artefacts. Table 1 summarises the measurement results regarding the tip and shaft artefact and the TLE.

**Table 1:** Artefact diameters and tip location error of 36 artefact measurements

	Sequence			Angulation			Orientation			Encoding direction		Total
	T1W-VIBE	T1W-FLASH	T2W-TSE	0°	45°	90°	Axial	Coronal	Sagittal	Phase	Frequency	
*A*_shaft_ (mm)	3.7 ± 2.4	6.3 ± 3.4	3.1 ± 1.8	1.4 ± 0.6	4.9 ± 2.1	6.7 ± 2.4	4.5 ± 2.3	4.0 ± 3.3	4.5 ± 3.3	4.3 ± 3.0	4.4 ± 2.9	4.4 ± 2.9
*A*_tip_ (mm)	1.3 ± 0.8	2.0 ± 0.9	1.7 ± 0.4	1.2 ± 0.8	1.6 ± 0.6	2.1 ± 0.5	2.1 ± 0.7	1.4 ± 0.6	1.4 ± 0.7	1.7 ± 0.8	1.6 ± 0.7	1.6 ± 0.7
TLE (mm)	-2.6 ± 1.4	-2.5 ± 0.2	-2.6 ± 0.8	-3.6 ± 0.6	-2.4 ± 0.5	-2.1 ± 0.8	_	-2.6 ± 0.9	-2.5 ± 1.0	-2.7 ± 0.8	2.4 ± 1.1	-2.6 ± 1.0

*A*_*shaft*_ Artefact diameter at the antenna shaft, *A*_*tip*_ Artefact diameter at the antenna tip, *TLE* Tip12 location error

**Fig. 2:**
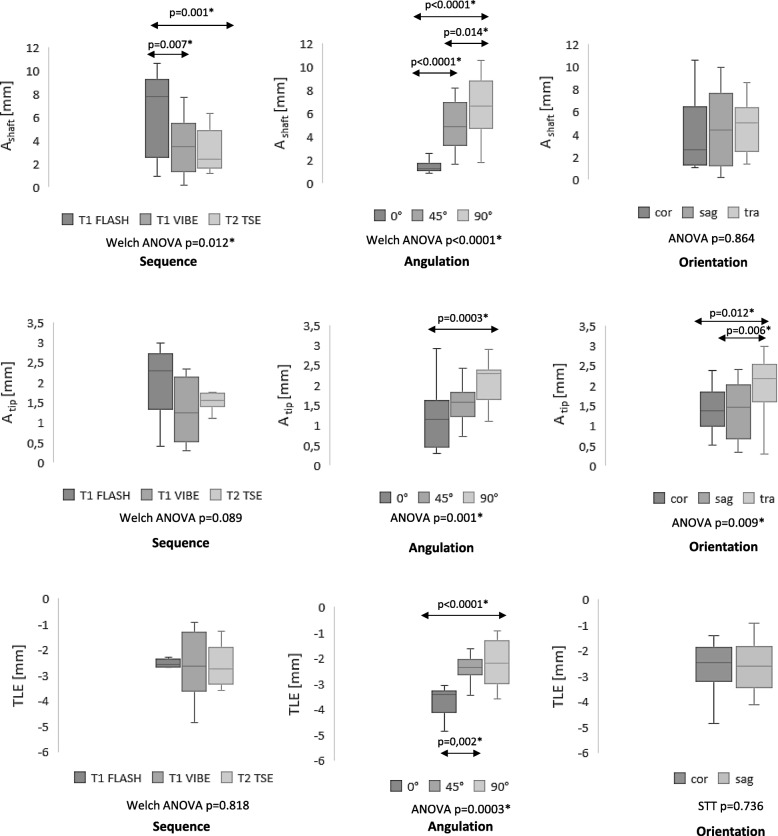
Statistical analysis regarding the influence of the sequence type, applicator angulation to *B*_0_ and slice orientation on the applicator artefact. Graphs display: *A*_shaft_ (artefact diameter at the antenna shaft), *A*_tip_ (artefact diameter at the antenna tip), and TLE (tip location error) of the microwave applicator in relation to sequence type, applicator angulation to *B*_0_ and slice orientation. Depending on < 2 or > 2 parameters Student’s *t* test (STT) or ANOVA was performed. In case of overall statistical significance, the Student *t* test was performed according to the closed testing procedure. Whiskers indicate the minimum and maximum extreme values; the box indicates the upper and lower quartile. The line in the box indicates the median value

**Fig. 3:**
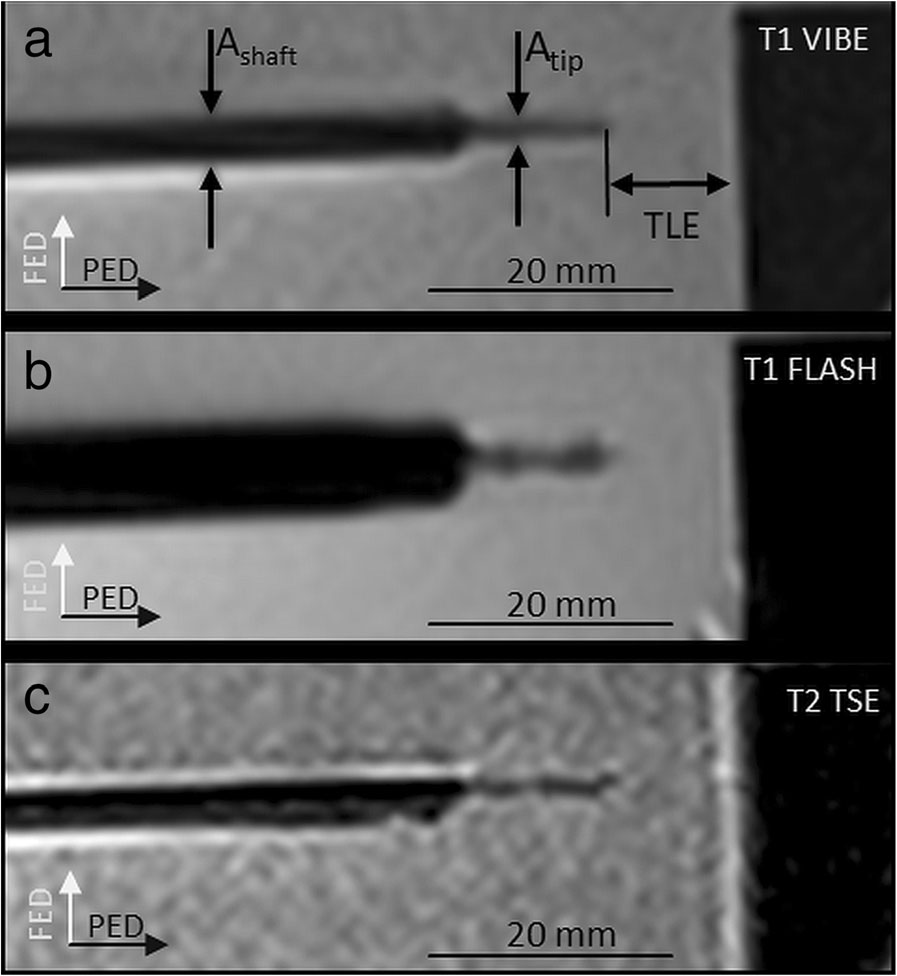
Artefact of the microwave applicator. Images are acquired under 90° applicator angulation relative to *B*_0_ with T1W-VIBE (**a**), T1W-FLASH (**b**), and T2W-TSE (**c**) (see text for sequence details). For all sequences, the applicator shows a similar artefact pattern with a prominent artefact at the shaft (*A*_shaft_) and a smaller artefact at the tip (*A*_tip_). *PED* Phase encoding direction, *FED* Frequency encoding direction

**Fig. 4:**
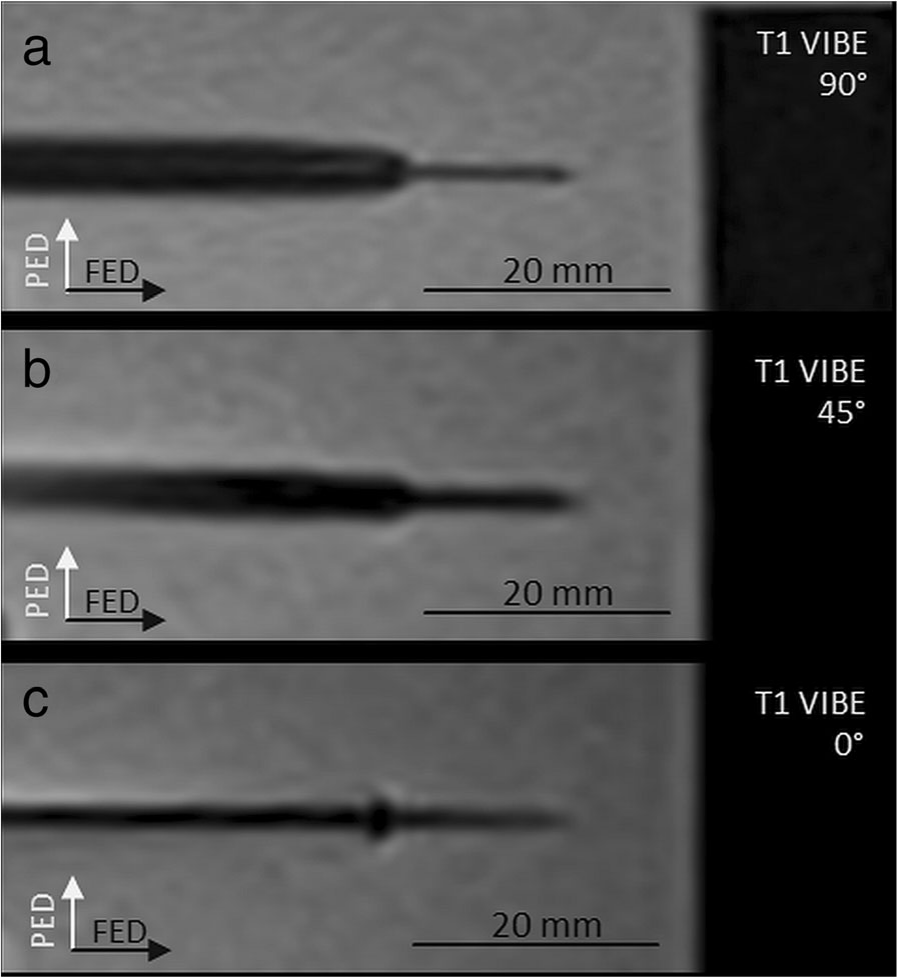
Artefact of the MW applicator according to the angulation to *B*_0_. Images are acquired with T1W-VIBE sequence (see text for sequence details) with an applicator angulation to *B*_0_ of 0°(**a**), 45°(**b**), and 90°(**c**). The diameter of shaft artefact was significantly dependent on the angulation with the smallest diameter with an angulation parallel to *B*_0_. *PED* Phase encoding direction, *FED* Frequency encoding direction

The mean diameter of the shaft measured 4.4 ± 2.9 mm with significant dependence on the sequence type (*p* = 0.012) and the applicator angulation to *B*_0_ (*p* < 0.001; Fig. 2). The significantly largest shaft artefact was measured with the T1W-FLASH sequence (6.3 ± 3.4 mm; Fig. 3). An applicator deflection from *B*_0_ orientation significantly increased the shaft artefact with a diameter of 1.4 ± 0.6 mm at 0° up to 6.7 ± 2.4 mm at 90° (Fig. 4).

The mean diameter of the applicator tip measured 1.6 ± 0.7 mm with statistically significant dependence on the angulation to *B*_0_ (*p* = 0.001) and the largest diameter for an applicator angulation perpendicular to *B*_0_ with a diameter of 2.1 ± 0.5 mm (Fig. 4). Largest tip artefact diameters were measured with the T1 FLASH sequence, however, without reaching statistical significance (*p* = 0.089). The largest statistically significant tip diameter was measured for axial slice orientation in relation to the applicator (*p* = 0.009) with a diameter of 2.1 ± 0.7 mm.

On average, the applicator length was slightly underestimated with a mean TLE of -2.6 ± 1.0 mm. The largest absolute TLE was measured for an applicator angulation of 0° (*p* < 0.001) with a TLE of -3.6 ± 0.6 mm.

The encoding direction did not affect the artefact diameter of the shaft (*p* = 0.935) and the tip (*p* = 0.492), and the TLE (*p* = 0.329).

### Ablation results

Ablation zone SAD and volume continuously increased with longer ablation durations and were significantly higher with the higher ablation power of 120 W. The highest mean ablation zone volume (56.78 ± 3.08 cm^3^) and mean SAD (42.0 ± 1.41 mm) was reached using an ablation power of 120 W and the longest recommended ablation duration (15 min). The SI ranged between 0.72 and 0.75 at the 5 min and 10min test series, without being significantly affected by the ablation power. With longer ablations (15 min), the sphericity of the ablation zone significantly decreased with the higher ablation power at 120 W (SI = 0.68 ± 0.05) in comparison to ablations at 80 W (SI = 0.75 ± 0.02; *p* = 0.048).

Table 2 summarises the ablation results regarding SAD, volume, and SI.

**Table 2:** Comparison of the 24 ablation results at 80 W and 120 W

	80 W	120 W	*p* value
5 min			
SAD (mm)	27.25 ± 2.63	32.0 ± 0.82	0.0136*
Volume (cm^3^)	14.75 ± 3.44	23.93 ± 2.47	0.0049*
SI	0.73 ± 0.08	0.72 ± 0.02	0.8324
10 min			
SAD (mm)	33.5 ± 1.29	38.0 ± 1.63	0.0050*
Volume (cm^3^)	26.45 ± 1.76	39.03 ± 3.69	0.0008*
SI	0.75 ± 0.05	0.74 ± 0.03	0.7835
15 min			
SAD (mm)	38.50 ± 1.91	42.0 ± 1.41	0.0259*
Volume (cm^3^)	40.15 ± 5.09	56.78 ± 3.08	0.0014*
SI	0.75 ± 0.02	0.68 ± 0.05	0.0480*

*SAD* Short axis diameter, *SI* Sphericity index

*Significant differences

## Discussion

Despite the energy transmission via a 4-m coaxial cable, the tested MR-conditional high-power MWA system reached ablations zones with a SAD above 4 cm within 15 min, which is comparable to high-power MWA systems designed for computed tomography (CT)-guided procedures [6]. Creation of sufficiently large ablation zones in a short time is relevant, especially to reduce intervention durations for MR-guided procedures. Rempp et al. [24] reported average procedure durations from planning imaging to control imaging of 3.7 h for MR-guided RF ablation in hepatic malignancies with a mean tumour diameter of 20 mm. These procedure durations are a major drawback of MR-guided interventions given the limited availability of MR scanners and higher costs of this modality in comparison to CT and ultrasound [25, 26].

The reasons for the long procedure durations in this study were the long net ablation durations of mean 52 ± 10 min and the need for applicator repositioning to treat a single tumour (mean 2.4 ± 1.3 applicator positions). Up to now, the expectation of shorter procedure durations under MR-guided MWA could not be fulfilled in clinical routine. Hoffmann et al. [27] recently reported procedures durations of 187 ± 64 min with a non-cooled, low-power MWA system with a maximum power of 36 W. Similar to the RF ablation study by Rempp et al. [24], multiple applicator repositioning (on average 2.5 ± 1.2) was necessary to treat the target tumours with a mean diameter of 15 mm. Further studies are necessary to evaluate if the promising *ex* *vivo* results of the tested high-power system can be confirmed *in* *vivo*, and if the relatively large ablation zones enable a reduction of the number of antenna repositioning procedures in clinical routine so that shorter procedure durations can be achieved.

Besides the volume and the SAD, the sphericity of the ablation zone is another relevant factor for fast and effective tumour ablation [28]. Several studies have shown that most spherical ablation zones were achieved in liver ablations using overlapping ablation zones with multiple antennas or repositioning of a single antenna [29, 30]. However, placement of multiple antennas or antenna repositioning is time-consuming and may increase the risk of complications due to the need for multiple liver capsule punctures. In our study, the SI ranged from 0.68 to 0.75. This value is similar to those reported for high-power MWA systems for conventional guidance techniques and higher than the SI value for a non-perfusion cooled MR-conditional system which has been reported to range from 0.36 to 0.59 [31].

Accurate and reliable visualisation of the artefact of the applicator is essential for a fast, safe, and effective tumour ablation. The appearance of the applicator artefact in MRI is predominantly dependent on the material and diameter of the applicator and the difference in magnetic susceptibility between the applicator and the surrounding tissue [32, 33]. In our study, the diameter of the applicator shaft artefact was significantly affected by the angulation to the main magnetic field. The largest diameter was measured with a perpendicular angulation to the main magnetic field, which is frequently applied during MR-guided percutaneous procedures in wide-bore scanners. The prominent artefact diameter at this angulation might be advantageous during applicator positioning. Nevertheless, smaller structures such as small target tumours could be obscured by the artefact, particularly if sequences are acquired which generate larger artefacts such as T1W-FLASH sequence that caused the largest artefacts in our series. However, the measured artefact diameter was smaller than the diameter of MR-conditional RF ablation applicators reported in earlier studies [21, 32]. The applicator tip artefact was clearly smaller than the shaft artefact. In our experimental study with ideal scanning conditions and a high contrast to the surrounding gadolinium solution, this tip area was well visible; however, the small tip artefact might be problematic especially if MR fluoroscopic sequences are used in patients with impaired respiration compliance. On the other hand, a small tip artefact may be beneficial during therapy monitoring. As reported in studies concerning MR-guided RF ablation using applicators with a larger tip artefact, retraction of the applicator can be necessary for evaluation of the target tumour and the ablation zone [24]. Small tip artefacts do not obscure the ablation zone and retraction of the applicator is not necessary for therapy monitoring. Consequently, in cases with an insufficient ablation zone, the ablation can be continued without the need for applicator repositioning [27].

Another essential point for safe applicator positioning is an accurate depiction of the applicator tip. According to the definition of TLE, a negative value implies an underestimation of the applicator length. This is relevant if the target tumour is located in front of critical structures and false depiction of the antenna tip can lead to an accidental puncture of the structure [34]. In our study, the length of the MW applicator was slightly underestimated with a TLE of -2.6 ± 1.0 mm. This TLE lays below a TLE of 5 mm, which was considered as a limit for MR-guided musculoskeletal interventions by Wonneberger et al. [35].

The results of our *ex* *vivo* study are limited by several factors. First, *ex* *vivo* ablations tend to overestimate the size of the ablation zone in comparison to *in vivo* ablations, due to the absent cooling effect of perfused tissue. On the other hand, we measured the size of the ablation zone after treatment. This zone is smaller than the untreated tissue, as the tissue shrinks during the ablation process, causing an underestimation of the ablated tissue [36]. Our artefact evaluation was conducted in a phantom under optimal conditions with a homogenous and high background signal without moving artefacts such as cardiorespiratory motion. *In vivo* studies are necessary to confirm the visibility of the device under clinical conditions.

In conclusion, the high-power MR-conditional MWA system tested in this *ex vivo* study provided a sufficiently dimensioned ablation zone suitable for tumour ablation. The artefact of the MW applicator seemed also suitable for MR-guided interventions with an accurate depiction of the applicator tip. These results are encouraging for the application of MR-guided MWA for percutaneous tumour ablation. However, clinical studies are necessary to confirm the potential benefit of the combination of both techniques.

## Abbreviations

FLASH: Fast low-angle shot
FOV: Field of view
LAD: Long axis diameter
MWA: Microwave ablation
RF: Radiofrequency
SAD: Short axis diameter
SI: Sphericity index
TE: Echo time
TLE: Tip location error
TR: Repetition time
TSE: Turbo spin-echo
VIBE: Volume interpolated breath-hold examination
